# Using Social Network Measures in Wildlife Disease Ecology, Epidemiology, and Management

**DOI:** 10.1093/biosci/biw175

**Published:** 2017-02-01

**Authors:** Matthew J. Silk, Darren P. Croft, Richard J. Delahay, David J. Hodgson, Mike Boots, Nicola Weber, Robbie A. McDonald

**Affiliations:** Matthew J. Silk (matthewsilk@outlook.com) and Robbie A. McDonald (r.mcdonald@exeter.ac.uk) are affiliated with the Environment and Sustainability Institute at the University of Exeter, in Penryn, Cornwall, United Kingdom. Darren P. Croft is with the Centre for Research in Animal Behaviour at the University of Exeter, in the United Kingdom. Richard J. Delahay is affiliated with the National Wildlife Management Centre of the Animal and Plant Health Agency at Woodchester Park, in Gloucestershire, United Kingdom. David J. Hodgson, Mike Boots, and Nicola Weber are with the Centre for Ecology and Conservation at the University of Exeter, in Penryn, Cornwall, United Kingdom; MB is also affiliated with the Department of Integrative Biology at the University of California, Berkeley.

**Keywords:** disease management, super-spreader, network metric, modularity, dynamic network

## Abstract

Contact networks, behavioral interactions, and shared use of space can all have important implications for the spread of disease in animals. Social networks enable the quantification of complex patterns of interactions; therefore, network analysis is becoming increasingly widespread in the study of infectious disease in animals, including wildlife. We present an introductory guide to using social-network-analytical approaches in wildlife disease ecology, epidemiology, and management. We focus on providing detailed practical guidance for the use of basic descriptive network measures by suggesting the research questions to which each technique is best suited and detailing the software available for each. We also discuss how using network approaches can be used beyond the study of social contacts and across a range of spatial and temporal scales. Finally, we integrate these approaches to examine how network analysis can be used to inform the implementation and monitoring of effective disease management strategies.

**Social structure is fundamental to the epidemiology** of the infectious diseases of humans (Newman 2002, May 2006) and animals (Craft and Caillaud 2011, Craft 2015, White et al. 2015). How individuals interact can influence how infection spreads through a population (May 2006, Cross et al. 2009, White et al. 2015), and how an individual interacts with others will affect its risk of being infected (Lloyd-Smith et al. 2005, White et al. 2015). For example, seasonal changes in social structure affect the disease dynamics of devil facial tumor disease in Tasmanian devils (*Sarcophilus harrisii*; Hamede et al. 2009), and differences among individuals in social relationships are correlated with bovine tuberculosis infection in European badgers (*Meles meles*; Weber et al. 2013). Social-network analysis (Croft et al. 2008, Krause et al. 2014) has transformed our ability to quantify and analyze population social structure in wildlife, especially alongside rapid technological developments in *biologging* (using animal-attached tags to log individual behavioral, physiological, or environmental data; Rutz and Hays 2009) that enable the automated remote monitoring of social interactions in an increasing range of species (Krause et al. 2013). However, a diverse array of analytical approaches fall within the scope of social-network analysis (see Croft et al. 2008, Farine and Whitehead 2015), and it can be unclear how these might best be applied to study and manage disease.

Here, we offer practical guidance on how to calculate and use social-network metrics to study disease ecology and epidemiology. Although the network tools described will be equally informative in the study of human disease (e.g., Rohani et al. 2010), we focus on their applications in animal populations, especially wildlife, because this is a rapidly developing field and because the practical applications for disease management are likely to be particularly valuable. Using network metrics to quantify individual-level and population-level patterns of social behavior and their relationship with epidemiological data not only provides an important descriptive and comparative tool but also yields valuable information for the statistical and epidemiological modeling of host–pathogen systems.

We first outline when social-network approaches are most relevant to epidemiological research. Next, we describe how network measures can be usefully applied, both for static and dynamic social networks. We then argue that network-based approaches are applicable beyond the study of social contacts or associations and can be creatively adapted to contribute to other aspects of epidemiological research (e.g., using networks of movements between geographical locations). Finally, we draw these ideas together to discuss briefly the potential utility of basic network tools in hypothesis testing and epidemiological modeling and to describe how quantifying these measures can be used by practitioners to inform strategies for the management of disease in wildlife populations.

## Social networks: The basics

**Figure 1.**
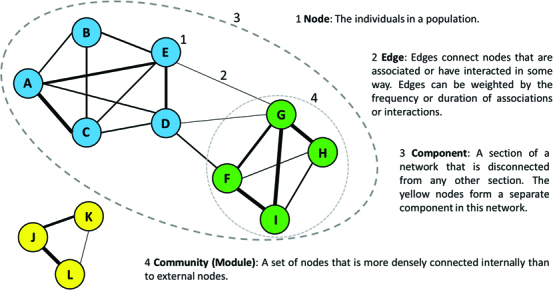
The basic components of social network structure.

Social networks represent the interactions of a population as a graph in which individuals are nodes or vertices and lines connecting individuals that have interacted are links or edges (figure 1). Edges can be weighted to represent the strength of an interaction and can either be directed (if the behavior has directionality; e.g., grooming behavior) or undirected. Social-network analysis (SNA) provides methods to quantify the patterns of social interactions in a population (figure 1; Croft et al. 2008, Pinter-Wollman et al. 2013, Krause et al. 2014), providing measures that describe the social structure of an entire (or sampled) population, as well as a wealth of information about the interactions of particular individuals. We direct readers new to SNA to a number of existing reviews for a general introduction (e.g., Croft et al. 2008, Pinter-Wollman et al. 2013, Krause et al. 2014, Farine and Whitehead 2015), and here, we focus on applications that are of particular value in wildlife disease research.

Edges in networks used for wildlife disease research should be defined with the disease being studied in mind. For example, the types of network or edge used to study directly transmitted parasites or pathogens would be different from those used for pathogens transmitted indirectly through the environment or perhaps via another vector. Furthermore, the type of association, behavioral interaction, or contact used to construct the network will be crucial to any inferences regarding disease transmission and therefore require careful selection by the researcher (Craft 2015, White et al. 2015). For example, when studying sexually transmitted parasites, it will be particularly important to consider networks of sexual interactions, perhaps moreso than those of intrasexual contests. If there is uncertainty over the likely modes of transmission, then SNA can be used to provide insights into the importance of these distinctions (direct versus indirect and interaction type).

Network data on animal social systems are typically collected using either observations of interactions or associations (Croft et al. 2008, Krause et al. 2014, Farine and Whitehead 2015) or biologging technology, such as proximity loggers or GPS loggers, to record proximity between individuals (Krause et al. 2013, 2014, White et al. 2015). For many disease studies, records of proximity or contact are sufficient, and the use of biologging technology is a preferred option (e.g., Hamede et al. 2009, Weber et al. 2013), because interactions between individuals are less likely to be missed. Network data can be stored as an *nxn* association matrix (where *n* is the number of individuals in the network) recording the frequency or duration of interactions among each dyad of individuals or as an edgelist containing information on the two individuals connected by each edge and the weight of that edge in separate rows for every completed edge.

## Network measures in static networks

In this section, we discuss the relative utility of different individual-level and population-level measures or metrics in static networks, which require less data and are easier to analyze than those in dynamic networks. We also provide practical guidance on how they can be calculated in R (R Development Core Team 2015) including a worked example.

### Measures of individual network position

Finding where individuals are located in a social network holds intuitive appeal as an approach to (a) understanding how important particular individuals are to the spread of infection and (b) understanding individual variation in infection risk. Individuals with many interactions act as hubs and have previously been described as *super-spreaders* (infected hosts giving rise to a disproportionately high number of secondary cases; Lloyd-Smith et al. 2005, Newman 2008), whereas others can act as bridges between different parts of a network (such as between two social groups) and may mediate the spread of infection (e.g., Weber et al. 2013). However, classifying individuals in this way has often previously used only one or two social network metrics, and these have varied among studies. Decisions on which metric to use are likely to depend on the questions being asked and the structure of the network in question; however, there have been no studies that have attempted to determine the optimum metrics for particular circumstances. This would be a useful area of future methodological research (box 1).

Box 1. **Where next for network methods to disease research?**

**Improved guidance on the best network measures to use**

Which network metrics best describe the risk of an individual acquiring infection and/or the importance of an individual in the onward spread of infection? How does this vary according to network structure?

**Understanding the implications of data constraints**

How do missing data affect the study of disease transmission using animal contact networks? Are there approaches that are robust to either missing individuals (nodes in the network) or missing contacts (edges in the network)?

**The use of (pseudo-)experimental approaches alongside observational studies**

Do disease-management strategies change social-network structure? Can we predict such changes using statistical models? Can using empirical network data inform evidence-based disease management?

**Using bipartite networks to determine indirect transmission**

Which metrics are most useful in bipartite networks? How effective is the comparison between bipartite-network data and contact-network data in determining indirect transmission? How are conclusions in bipartite networks affected by missing data?

**Figure 2.**
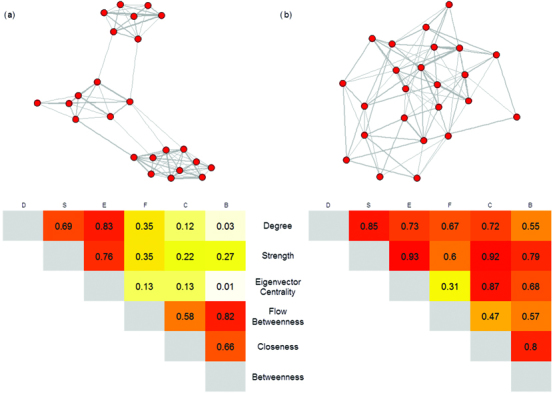
The correlations between centrality metrics for (a) a network with high modularity and (b) a network with low modularity containing the same number of nodes. Correlations are calculated using the Pearson correlation coefficient, with shading showing the strength of the correlation (darker colors represent stronger correlations).

Measures of centrality (degree, strength, eigenvector centrality, flow betweenness, betweenness, and closeness) are typically the most directly relevant metrics to disease research because they measure key aspects of an individual's connectivity or importance to overall social structure (see table 1). These metrics lie along a spectrum from local to global measures of network position (and are ordered as such below), with the latter accounting for the structure of the whole network. Applications of these centrality metrics to disease research will vary according to their position on this spectrum. They are often correlated in well-connected networks but can describe very different network positions in which populations exhibit more substructure (figure 2; Farine and Whitehead 2015). We take this into account when discussing the application of these centrality metrics to disease research below (the details on what they measure are in table 1).

**Table 1.** A summary of the key network metrics used in disease research and how they are most usefully applied.

Metric	Individual-level or population level	What does the metric measure?	R package and function
Density	Population	The proportion of completed edges in the network	igraph – edge_density()
						sna – gden()
Mean path length	Population	The mean of the distance in steps through the network between all possible pairs of individuals	igraph –
						mean_distance()
						tnet – distance_w()^a^
						sna – geodist()^a^
Transitivity	Population	The amount of clustering in the network, as is calculated as a function of completed triangles (A being connected to C, when A is connected to B and B to C) relative to possible triangles	Unweighted:
						igraph – transitivity()
						sna – gtrans()
						Weighted:
						tnet – transitivity_w()
Degree	Individual	The number of connections an individual has in the network	tnet – degree(w)
Strength	Individual	The combined weight (i.e., frequency or duration) of all of an individual's connections in a network	igraph – degree()
						tnet – degree_w()
Eigenvector centrality	Individual	A measure of influence in the network that takes into account second-order connections (i.e., connections of connections)	igraph – eig
						en_centrality()
Closeness	Individual	A measure related to the normalized mean path length from that individual to all other individuals in the network	tnet – closeness_w()^b^
Betweenness centrality	Individual	The number of times a node (individual) occurs on the shortest path between two other nodes in the network	tnet_closeness_w()^b^
Flow betweenness	Individual	A second measure of betweenness centrality that measures the centrality of an individual as a function of the “flow” through it rather than purely with respect to shortest paths	sna – flowbet()

a These functions calculate a matrix of all path lengths. A mean would then need to be calculated.

b Suggested over the equivalent igraph functions because of how edge weights are incorporated (see main text).

*Degree* is the number of connections an individual has in a network. Individuals with high degree are more likely to be exposed to infection during an epidemic and will have the opportunity for onward spread of infection to a greater number of individuals.

*Strength* also takes into account the weight of an individual's interactions (i.e., the frequency or duration of contact events) and is therefore likely to be more informative than degree when the risk of transmission increases as individuals spend more time interacting or interact more frequently. As a result, using strength rather than degree will be of greater relevance for pathogens with low infectiousness. A key weakness of both strength and degree is that they are local metrics that only take into account the immediate neighborhood of a given individual. This may potentially limit their value for investigating the spread of infection, especially in networks where distinct substructure means that some connections are more important than others.

*Eigenvector centrality* is the second-order connectivity of an individual, and similarly to strength and degree, individuals with higher eigenvector centrality are likely to be at a higher risk of exposure to infection and to be potentially more important for the onward spread of infection, especially locally. Although eigenvector centrality is a less local metric of network position than strength or degree, it typically describes a similar social-network position in networks with distinct substructure (figure 2); therefore, this metric has somewhat similar applications and limitations to degree and strength.

*Closeness* is a global metric and will be important in determining the risk of exposure of an individual during an epidemic, especially in networks with greater substructure because individuals with high closeness tend to have connections that span between different modules (figure 1) or social groups.

*Betweenness* (we describe *Freeman Betweenness centrality*) is a global metric that is particularly valuable for measuring the importance of individuals in connecting different parts of the network. This makes it a useful metric to consider when describing the ability of an individual to mediate the spread of infection during an epidemic, especially in networks with considerable substructure (e.g., social groups).

*Flow betweenness* is also a global measure, but it provides a metric that is somewhere between strength and betweenness (figure 2, supplemental table S1). As it describes flow through the network, it is especially pertinent to studying disease spread. This may also make it particularly relevant to determining the risk or likelihood of an individual becoming infected and the role of an individual in regulating spread of an endemic disease within a population (i.e., a “spread-capacitor”; Weber et al. 2013). It is also better at capturing the importance of particular interactions in networks with greater substructure (e.g., interactions that occur between social groups) than more local centrality metrics (figure 2).

In general, in well-connected networks (with high edge density) with limited substructure, the choice of metric is relatively unimportant (figure 2). However, in more subdivided networks (e.g., where individuals are found in social groups), there is an important distinction between local and global metrics. Local metrics (e.g., degree, strength, and eigenvector centrality) are more likely to capture the short-term exposure of individuals to infection, especially when nearby individuals are already infected. However, to capture the importance of individuals in spreading infection, especially in identifying *spread-capacitors* (individuals involved in regulating the spread of infection between network components), it is important to consider more global metrics, particularly betweenness and/or flow betweenness. Furthermore, the subtle distinctions between global metrics mean that they describe network positions that have different effects on disease transmission, and these distinctions could be fundamental in informing use of individual-level metrics in networks with different structures. For example, when network substructure is intermediate, individuals with high flow betweenness are more likely to control or mediate disease spread when the disease is endemic (e.g., Weber et al. 2013), whereas betweenness may better identify individuals with the potential to be super-spreaders during epidemics. However, in highly substructured networks, betweenness and flow betweenness are likely to be closely correlated ­(figure 2).

### Population-level measures of network structure

Network-level metrics can be usefully applied to the study of disease epidemiology in wildlife populations (table 1). These metrics can help describe the susceptibility of a population to disease and the rate at which epidemics might spread through it. What they measure in terms of network structure is outlined in table 1.

*Edge density* is the proportion of completed edges (i.e., observed interactions) in the network, and disease transmission would be expected to occur more rapidly in networks with higher edge density. Edge density alone would be sufficient to describe the susceptibility to epidemic spread in networks with limited substructure because it will describe typical interaction frequencies in the population, but it is insufficient to describe the susceptibility of more substructured networks, where there is greater heterogeneity in interaction frequency.

*Average path length* would be expected to be lower in networks with a higher density of edges or reduced substructure, such that lower average path length would be expected to be associated with faster spread of infection.

*Transitivity* can be useful in providing an idea of network substructure. For example, lower-density networks with high transitivity are likely to be more subdivided into different modules and therefore are likely to be less susceptible to disease spread.

Population-level metrics are especially useful in combination with one another and with individual-level metrics expressed as population means and coefficients of variation. This is particularly true for the detection of substructure or subdivisions within the overall network structure. For example, networks with high variance in centrality metrics, especially betweenness, are likely to contain more substructure. This is important because in these populations, we would expect infected hosts to be more aggregated and the spread of infection to be relatively slow and more dependent on the traits of particular individuals (e.g., super-spreaders or spread-capacitors).

### Software

All the metrics discussed above can be calculated in R (R Development Core Team 2015) using the packages sna (Butts 2008), igraph (Csardi and Nepusz 2006), and tnet (Opsahl 2009). The most useful functions are shown in table 1, and we demonstrate their use in our worked example (box 2, supplemental material). The package igraph offers the best plotting options to initially depict networks and facilitates the calculation of many of the above metrics in weighted networks. However, sna is required to calculate flow betweenness. In tnet, it is also possible to calculate weighted network metrics. An important difference between tnet and igraph is that in tnet, edge weights are (by default) treated as a benefit when calculating weighted metrics, whereas in igraph, by default, they are treated as a cost. The assumption used by tnet is the more appropriate one for wildlife disease research. Another advantage of analyzing weighted networks using tnet is the ability to use an alpha parameter in network metrics to control the importance of interaction weights in the metric. This could be of interest to researchers looking to explore the relative importance of interaction weight on disease transmission.

Box 2. **Social network analysis of European badgers.**

Here, we provide a worked example of network analysis in a wild animal population using data from Weber and colleagues (2013). The data in this study were collected using proximity loggers deployed on 51 individuals in a UK population of European badgers (*Meles meles*) naturally infected with bovine tuberculosis (for more details on the methods, we refer readers to the original study).

**Figure 3.**
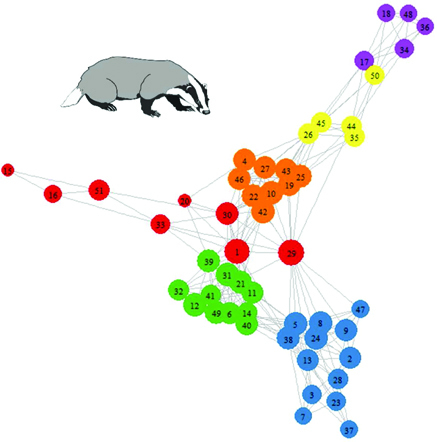
A contact network of badger social interactions. Nodes are colored by social community membership as was determined by the fastgreedy algorithm applied in igraph. Node size is related to the flow betweenness of the individual; those with greater flow betweenness are represented by larger nodes. Badger image obtained under a Creative Commons license from http://animalsclipart.com/stencil-badger-clipart-design.

We provide R code demonstrating how to calculate the individual-level and population-level network metrics discussed in this article (see table 2), plot the network, and calculate its community structure and modularity (see supplemental material). The badger population has a social network with high modularity and six cliques or communities detected (*Q* = 0.75 for this subdivision). Modularity structure is driven principally by association with a main sett (the communal burrows used by territorial social groups) and is illustrated by node color in figure 3. There is also considerable individual variation in centrality in this network (table S1), and this is demonstrated by the size of the nodes in figure 3.

**Table 2.** Values for population and individual-level social network metrics calculated in a contact network of wild European badgers. The mean and variance of individual-level metrics are also provided.

Metric	Population-level or individual-level metric	Value (mean for individual-level metrics)	Variance (for individual-level metrics)
Density	Population	0.19	NA
Average Path Length	Population	2.48	NA
Unweighted transitivity	Population	0.57	NA
Weighted transitivity	Population	0.71	NA
Degree	Individual	9.69	16.70
Strength	Individual	314031.9	58335024920
Eigenvector centrality	Individual	0.42	0.07
Closeness	Individual	4.23 ⋅ 10^–6^	2.10 ⋅ 10^–12^
Betweenness	Individual	108.27	35484.96
Flow betweenness	Individual	2957938	1.57 ⋅ 10^13^

**Figure 4.**
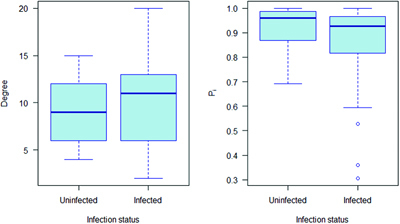
The degree (number of connections) and participation coefficient (Pi; proportion of connection within their own network community) for European badgers in relation to bovine tuberculosis infection status as defined by Weber and colleagues (2013). The solid line represents the median, the box the interquartile range and the whiskers extend 1.5 times above and below the upper and lower quartiles respectively.

The relationship between network position and infection is complex in this population, with infected individuals tending to form more out-of-group contacts and fewer within-group contacts at particular times of year (Weber et al. 2013). Nevertheless, there is an overall trend for bTB-infected individuals to have higher-degree (i.e., more contacts) and lower P_i_ (i.e., more out-of-group contacts), as would be expected from these results (figure 4).

By using the information in figure 3 and supplemental table S2, it would be theoretically possible to identify the individuals most likely to spread infection through the network, raising the possibility that such individuals could be targeted by management interventions. For example, individual 29 has the highest values of flow betweenness and degree and the lowest value of P_i_ and so may be pivotal in facilitating disease transmission through the network.

## Modularity approaches in static networks

The substructure of networks can be determined using modularity approaches. A *modularity score* is a measure of the strength of division of a network into different modules, such as communities, clusters, and social groups (Girvan and Newman 2002, Newman and Girvan 2004, Newman 2006). Modularity scores can be used as a tool to compare the structure of different networks (either from separate populations or from different sets of interactions within the same population), especially in populations that consist of relatively stable social groups. Modularity-based approaches can also provide an alternative means of assessing the relationship between network position and infection in a way that is directly linked to any structure or social system present in the population (e.g., stable social groups) and will therefore complement approaches using network metrics.

### Using network-level modularity approaches

At a network level, modularity can have important implications for the spread of disease, because networks with considerable substructure and therefore higher modularity scores are likely to be less susceptible to the rapid spread of infection and less likely to conform to the assumption of random mixing used in many disease models (Cross et al. 2009). Furthermore, the modularity of network structure will influence the implications of different network positions for disease spread. In networks with higher modularity, the added importance of connections among modules or communities (figure 1) will mean that more global measures would be expected to be more informative of a high number of secondary cases (e.g., compare figure 2a and figure 2b: the network in figure 2a has higher modularity).

### Extending modularity approaches to an individual level

There are various individual-level metrics that rely on module assignment (see Guimera and Amaral 2005). The most relevant to working out an individual's potential role in disease spread are P_i_ (the proportion of an individual's interactions with individuals from the same versus different modules) and z_i_ (a normalized measure of the strength of an individual's interactions within its module), and the formulae to calculate these are detailed below. Individuals with high z_i_ will be likely to play an important role in spreading infection within a social group or local region of the network. Individuals with low P_i_ are likely to be more important for disease spread through the wider population (either in a super-spreader or spread-capacitor role), because they will be responsible for the majority of intermodule interactions that allow epidemics to spread through a structured social network.

(1) \begin{equation*}{z_i} = \frac{{{D_i} - {{\bar{D}}_{{s_i}}}}}{{{\sigma _{{D_{{s_i}}}}}}} \end{equation*}

*D_i_* is the number of within-module connections, ${\bar{D}_{{s_i}}}$ the mean number of within-module connection for that module, and *σ_D__S__i_* the standard deviation around this mean.

(2) \begin{equation*}{P_i} = 1 - \sum\nolimits_{S = 1}^{{N_M}} {{{\left( {\frac{{{D_{is}}}}{{{K_i}}}} \right)}^2}} \end{equation*}

D_is_ is the number of within-module connections, and K_i_ is an individual's overall degree.

### Software

Of the R packages introduced previously, igraph offers the widest range of algorithms for module or community detection. The edge-betweenness method for community detection using the Girvan-Newman algorithm (Newman 2004) has been widely used in the past (e.g., Lusseau and Newman 2004, Lusseau et al. 2006, Manno 2008); however, two more recent algorithms (Fast-Greedy and Multi-level, both implemented in igraph) are now often preferred as they are more time efficient and can be used with weighted networks. Besides igraph, more sophisticated methods of community detection can facilitate greater uncertainty in membership, which may be of interest in some situations. For example, it is possible to incorporate uncertainty through the “soft” assignment of individuals into communities with a probability or level of confidence (e.g., Lusseau et al. 2008) or by incorporating an individual in multiple communities (e.g., Palla et al. 2005, Psorakis et al. 2011). P_i_ and Z_i_ (and other metrics related to modularity) can be calculated using formulae provided by Guimera and Amaral (2005). We provide an example of community detection and the calculation of Z_i_ and P_i_ in box 2 and the supplemental material.

## Extending network measures to dynamic networks

**Figure 5.**
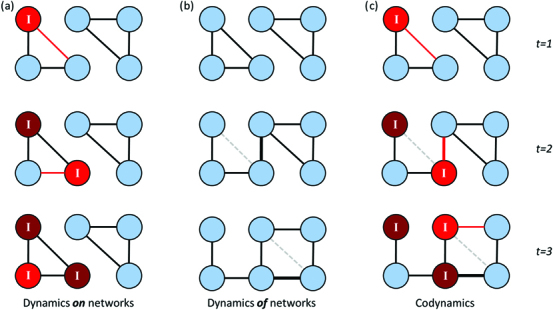
The complex dynamics of disease and social behavior displayed as (a) disease dynamic on a network, (b) social network dynamics, and (c) disease dynamics on a changing social network in a toy network over three time steps. In (a) and (c), the red nodes are infected and marked “I”, and the red/mid-gray edges represent transmission in that time step. The lighter red nodes have just become infected in that time-step. In (b) and (c), one edge is changed in between each time step, with the edge that is lost marked as a dashed light gray line in that time step.

Incorporating a dynamic view of population social structure (instead of the static network approaches discussed so far) will add substantially to the applications of social networks to wildlife disease epidemiology (figure 5). This is because patterns of social interactions are inherently dynamic, and their order matters for the acquisition and transmission of infection. Similarly, changes in infection status may influence interactions with other individuals. This results in social structure and infection covarying (figure 5; White et al. 2015) and means that using metrics to describe the dynamic features of the networks can provide additional insights into disease spread. Temporally dynamic metrics can describe either time-aggregated network snapshots in which temporally explicit data are collapsed into a series of static networks or temporally explicit time-ordered networks in which the temporal information is retained alongside the interaction data.

### Network snapshots

Studies in Tasmanian devils (Hamede et al. 2009), European badgers (Weber et al. 2013), and raccoons (*Procyon lotor*; Reynolds et al. 2015) have all been used to link seasonal variation in contact-network structure to the infection status of hosts. In each of these studies, a network snapshot approach was adopted whereby contact networks were aggregated over particular time windows (climatic seasons: Weber et al. 2013, Reynolds et al. 2015; reproductive seasons: Hamede et al. 2009). Hamede and colleagues (2009) explored seasonal variation in network structure and linked this to the susceptibility of the population to disease spread during different periods, whereas Reynolds and colleagues (2015) took this a step further by modeling epidemics on networks at different times of the year. The study by Weber and colleagues (2013) was more individual based and explored the relationship between bovine tuberculosis infection in badgers and network position at different times of year. Using serial network snapshots calculated at appropriate intervals enables researchers to use the same set of metrics used in static networks, and we refer the reader to the previous section.

### Dynamic network metrics

Time-ordered networks account for the precise order of interactions in a population. This level of temporal information is now more widely available, because wildlife networks are increasingly constructed using data from proximity loggers (e.g., Hamede et al. 2009, Weber et al. 2013, Blyton et al. 2014). These devices offer much potential in the generation of animal-contact networks if variation in performance is correctly accounted for through pre-deployment calibration or post-deployment corrections (Drewe et al. 2012). Two useful dynamic network metrics for disease research are the shortest time path and spread analysis.

*Shortest time path* is the shortest path in time between an individual and any other individual in the population. At an individual level, shortest-time-path lengths may help highlight individuals that are likely to play a key role in disease transmission and provide an indication of whether they maintain these network positions over time or achieve them by displaying highly dynamic social associations. Therefore, by taking the order of events into account, such metrics could help clarify how super-spreaders emerge and provide a more temporally explicit idea of the consequences for disease spread.

*Spread analysis* is the number of unique nodes that can be reached from an individual or set of individuals in a given time window. A measure of spread analysis during a particular period would provide an indication of the maximum possible rate at which an epidemic could spread through a population during that time. Temporal changes in this metric could provide an indication of when populations are most susceptible to rapid disease spread. Similarly, variation in the outputs of spread analysis for different regions of the network (i.e., between different parts of the population) or over time could be correlated with changes in the prevalence or incidence of infection at smaller spatiotemporal scales to better understand how the ordering of interactions during a given time period can contribute to the spatial aggregation of infected hosts.

### Software

The use of time-ordered networks is discussed in Blonder and colleagues (2012), and the associated R package timeordered enables basic analysis (including calculation of the above metrics). In addition, it is possible to convert time-ordered networks to time-aggregated networks (or snapshots; see above) and perform randomizations that may be required for hypothesis testing (see section below).

## Getting creative with network approaches

In this section, we discuss three ways in which network analytic approaches could be applied outside the study of social contacts to provide insights into disease, using (1) multiple network approaches to understand how transmission of infection occurs, (2) network analysis to explore site connectivity and disease epidemiology at large spatiotemporal scales, and (3) network analysis in long-term data sets to uncover long-term trends in population structure. There will be other novel ways in which network methodologies can be applied to the study of wildlife disease, and we encourage researchers to think creatively as to how they might apply network approaches in this field.

### Using networks to understand how transmission occurs.

Using multiple types of network simultaneously can facilitate the identification of the social contacts or types of behavioral interactions that are most important in disease spread and may permit estimation of the relative importance of direct and indirect transmission. Individual- and population-level metrics can then be used to compare the relationship between networks and disease in the different constructed networks.

To establish the role of different types of social behavior in disease transmission, separating networks by type of behavioral interaction can reveal the relative importance of particular behaviors. For example, in mountain brushtail possums (*Trichosurus cunninghami*), strain-sharing of *E. coli* has been shown to be more closely linked to networks of nocturnal interactions than to networks based on diurnal den-sharing (Blyton et al. 2014). Networks can also be split by the type of individuals interacting. It might be predicted, for example, that biases in disease acquisition between the sexes are related to differences in male–male, female–female, and male–female contact networks (e.g., Hamede et al. 2009). Sex- and age-related variation in pathogen acquisition may be fundamental drivers of population-level disease dynamics.

In order to describe the indirect transmission of a pathogen through a population, one needs to construct both a social network and also a bipartite network. Bipartite networks link individuals to spatial locations of importance (e.g., resting locations, foraging patches, and latrines). They are most useful when there are pre-existing hypotheses about environmental parasite transmission. For example, if the use of latrines is thought to be especially important in transmission, then the use of bipartite networks that link individuals that use a latrine within a particular time interval would be expected to predict which individuals acquire infection. Bipartite networks can then be collapsed to form an equivalent one-mode network according to specific criteria. For example, Godfrey and colleagues (2009) showed that in gidgee skinks (*Egernia stokesii*), a network based on refuge sharing during the estimated transmission period was a better predictor of tick and blood parasite transmission than networks based directly on social associations. There is plenty of scope to develop this approach further. Incorporating different time lags when setting the criteria to collapse the bipartite network, for example, could help identify the infective period of a location when a pathogen is transmitted indirectly via the environment. Similarly, considering multiple networks that distinguish space sharing in a range of environments (e.g., dens, foraging sites, and latrines) could enable the identification of important behaviors for disease spread via the environment and better assess how the risk of exposure to a pathogen varies across the landscape. The latter in particular requires high-resolution biologging data that have only recently become available (Krause et al. 2013) and is therefore likely to become increasingly feasible in animal systems. The use of approaches using bipartite networks in this way remains relatively untested, and its ability to separate direct and indirect transmission and its susceptibility to missing interactions require further investigation (box 1).

### Network analysis, spatial connectivity, and epidemics.

Using networks to quantify population connectivity on large spatial scales is another useful application of network analysis, as is illustrated by its use to study the role of livestock movements in the spread of disease (e.g., Christley et al. 2005, Kao et al. 2006, Kiss et al. 2012). This approach may be particularly powerful for investigating the role of dispersal and migratory behavior in epidemics of wildlife. Many migratory species travel huge distances and can be instrumental in moving infection between widely separated areas (Hoye et al. 2011). Using networks to quantify spatial connectivity could help us to predict disease spread among migratory flyways and species (e.g., avian influenza: Chen et al. 2005, Hoye et al. 2011).

Both conventional network metrics and modularity approaches could be useful for analyzing networks that use locations rather than individuals as nodes. Measures of centrality could be applied at this scale to calculate whether particular locations contribute disproportionately to disease spread, in practice functioning as “super-spreader locations.” In many migratory populations, these might be expected to be hubs or migration bottlenecks (Silk et al. 2014). Modularity approaches could help identify clusters of sites that are closely linked and therefore at greater risk if infection emerges in an area. P_i_ could additionally be used to identify sites that are important in connecting different clusters of associated sites. Therefore, being able to use both conventional network metrics and modularity approaches can provide important information to aid disease risk modeling and management on these larger spatial scales.

### Network analysis to link long-term trends in the social, demographic, and epidemiological structure of populations.

Long-term studies allow us to describe the role of disease in individual survival (e.g., McDonald JL et al. 2014) and the subsequent demographic consequences (e.g., Lachish et al. 2009, Wobeser 2013, McDonald JL et al. 2016), and this can be key in improving our understanding of wildlife disease ecology, especially for chronic, endemic infections (e.g., McDonald JL et al. 2014, 2016). However, there has been very little research exploring how long-term trends in demographics, population social structure, and disease are linked. Studies of mixed-species flocks of tits (*Paridae spp.*) in Wytham Woods, United Kingdom, illustrate the power of integrating multigenerational social networks with a longitudinal study (e.g., Aplin et al. 2013, 2015, Farine and Sheldon 2015). Although most long-term data sets may not include fine-scale interaction data, in social species, they often include information on social-group membership or site use (particularly feeding or resting sites). This can be used to quantify a population social structure via a bipartite network that links individuals that have used these sites within a given time window. Although this does not provide direct information on interactions or contacts, it does enable broader-scale trends in population structure to be identified and quantified (e.g., the dispersal of individuals between social groups). Furthermore, using this approach for network construction increases the feasibility of constructing multigenerational networks over extended timescales and facilitates their integration with demographic processes and individual life histories. For example, changes in social structure could be linked to environmental changes, demographic trends, or dispersal. Events such as these may be important in driving changes in social-network dynamics that facilitate phase shifts in disease epidemiology. Furthermore, information on the social behavior of individuals would be available over much of their lifetime and could be directly related to changes in infection risk or disease susceptibility (e.g., mediated through variations in senescence rates, condition, and stress). Therefore, not only does applying network analysis in this way negate the need for additional cost- or time-intensive fieldwork, but it also provides a stronger link with demographic processes.

## Network metrics in hypothesis testing and epidemiological modeling

Calculated network metrics can be used to test hypotheses related to network position (Croft et al. 2011, Farine and Whitehead 2015) or, alternatively, to help parameterize epidemiological models (Craft 2015). We discuss this in relation to social networks, but it will be equally applicable to spatial or bipartite networks.

Testing hypotheses related to network metrics is the principal means of linking disease status and other individual traits with social network position. However, hypothesis testing with social-network data is not a trivial undertaking, owing to the relational (nonindependent) nature of the data, especially at an individual level. The network metrics calculated for any given individual rely on the metrics of other (nearby) individuals and therefore are nonindependent, meaning that tests of significance require the use of network randomizations (see Croft et al. 2011, Farine and Whitehead 2015). Randomization approaches generate uncertainty around the null hypothesis by permuting the data used to construct the observed network. This makes it possible to test the statistical significance of features related to the observed network. Network randomizations may also offer a way of reducing network edge effects, especially if spatial information is included. However, finding an appropriate method of randomizing a network is vital to drawing the correct conclusions and the randomizations used depend on the study system (Croft et al. 2011). Further information on the design and implementation of randomization or permutation procedures is available in Croft and colleagues (2011) and Farine and Whitehead (2015). For interaction-based networks (likely to be widespread in disease research), it can be sufficient to perform swaps of node labels (individual traits such as sex or disease status) or edges (possible using various algorithms in social network packages in R such as igraph (Csardi and Nepusz 2006). For association-based networks, it is often necessary to randomize the original data set, and this can be done using the R package asnipe (Farine 2013).

For epidemiological models, metrics can be used to provide parameters for the generation of networks when modeling disease and also to check the goodness-of-fit of already simulated networks to those measured empirically. Knowledge of properties such as degree distribution (the histogram or density plot of individual degree), edge density, and network modularity can be used to simulate networks that are very similar to the observed population. An excellent example of this approach was provided by Hamede and colleagues (2012), who used sex-specific association rates from previously constructed social networks alongside a parameter that varied the level of clustering in the network to estimate seasonal contact patterns. The same properties, especially degree distributions and measures of clustering (e.g., a triad census), are typically used as goodness-of-fit tests when simulated networks for modeling are generated using other methods (e.g., exponential random graph models in Reynolds et al. 2015).

Both statistical and mathematical modeling of contact networks and disease offer significant opportunities to improve our understanding of how networks play a role in disease transmission in wildlife populations. However, further work is required to develop the necessary methods. Social and epidemiological data from wild animal populations can suffer from missing data issues (Craft 2015), and determining which approach is more robust to this problem in different contexts is a critical methodological challenge (box 1).

## Using network metrics to inform and monitor disease management

The management of disease in wildlife populations can involve targeting of the infectious agent (using vaccination or treatment), targeting the host population (by culling), or manipulation of the environment (Delahay et al. 2009). We outline in this section how network metrics can be used to provide insights into disease systems in wildlife that may help inform and direct such interventions.

Interventions to manage disease in wildlife populations can be fraught with uncertainty regarding likely outcomes. Culling host populations, in particular, has been associated with both positive and negative impacts on disease control (Macdonald 1995, Donnelly et al. 2006, Holmala and Kauhala 2006, Streicker et al. 2012), and environmental manipulation for disease control has been associated with catastrophic impacts on wildlife populations (Mbaiwa and Mbaiwa 2006). Social-network-analysis approaches offer a means of understanding the pre-existing social structure in populations that may be subject to interventions, as well as of identifying the significant changes in contact patterns that may result from demographic and behavioral responses to interventions and that may give rise to counterproductive epidemiological outcomes. Such information may be useful in designing interventions so they minimize counterproductive effects and maximize cost-effectiveness. For example, information on the prevailing contact structure of host populations may allow the refinement of vaccination, culling, or treatment interventions such that they target groups, areas, or time periods that contribute disproportionately to disease spread. Furthermore, it may even be possible to use social-network data directly to identify individuals to target for intervention. For example, hypothesis testing could be used to link the characteristics of individuals to the probability that they would be located in high-risk network positions, and then the correlated individual attributes could potentially be used to target individuals for management interventions.

A similar approach can be used with multiple network types or spatial networks to target environments and modes of transmission for effective disease control. Spatial networks can be used to target sites in which controlling disease may have the greatest impact. For example, interventions in sites with high flow betweenness or P_i_ in a spatial network may be particularly beneficial. Using multiple network types may permit identification of the most likely modes of transmission and targeting of the environment through which these interactions occur. For example, the finding of Blyton and colleagues (2014) that *E. coli* transmission in brushtail possums was more closely linked to nocturnal encounters than den sharing would suggest that (if this was a pathogen subject to management) control mechanisms targeted at shared refuges would not be as effective as might intuitively be expected.

Network metrics could also be used to assess the potential consequences of management interventions (see box 2 for an example). Their effectiveness will depend on how social interactions respond to the management strategy used. For example, some culling approaches disrupt population social structure and alter contact networks in a way that can increase disease transmission (McDonald RA et al. 2008), and this could be monitored by measuring reductions in modularity or average path length. At an individual level, the effectiveness of targeting individuals in particular network positions with management interventions depends on how social networks respond dynamically to them. For example, if individuals that are treated or vaccinated against an infection change their social behavior and no longer occupy high-risk network positions, the targeting of these individuals is less effective. Similarly, if the culling of these individuals reduces the connectivity of the overall network, then this management strategy will be far more effective than if other individuals occupy similar network positions when the original individuals are removed. If high-resolution network data (e.g., using biologgers) from before, during, and after a management intervention are available, then dynamic network metrics could be used to quantify the consequences. For example, the shortest time path and spread analysis calculated over various time windows during a management intervention would describe how the rate of spread of infection through the population is responding.

## Conclusions

Network analysis offers an invaluable toolkit for those studying and managing disease in wildlife populations. A range of modeling approaches specific to networks are available, but they are still rarely applied in wildlife disease management and require further development to enhance their potential value. By highlighting the utility of a more basic approach using descriptive network metrics, we hope to facilitate the use of network approaches by researchers and practitioners not familiar with network analysis. This has the potential to enable more effective management of some wildlife diseases, as well as triggering further research on the use of social networks to inform and study disease management in wild animals.

## Supplementary Material

Supplementary Data
