# Supplementary material for: Using Social Network Measures in Wildlife Disease Ecology, Epidemiology, and Management
Source: Bioscience. 2017 Feb 1;67(3):245–57. doi: 10.1093/biosci/biw175 (PMC5384163; doi:10.1093/biosci/biw175)
Supplement: Supplementary Data [file biw175_Supplementary_Data.zip › biw175_Suppp.zip!/Supplementary example.docx]

**Supplementary example results**

Table S2. Values for individual-level social network metrics calculated in a contact network of wild European badgers. Closeness values have been multiplied by 10^6^. NA values for unweighted Z_i_ occur when all individuals in a social community have equal degree.

| **Individual** | **Degree** | **Strength** | **Eigenvector centrality** | **Closeness** | **Betweenness** | **Flow betweenness** | **Z_i_** | **Unweighted Z_i_** | **P_i_** | **Unweighted P_i_** |
| --- | --- | --- | --- | --- | --- | --- | --- | --- | --- | --- |
| 1 | 20 | 495163 | 1.00 | 5.16 | 828 | 18374376 | 1.09 | 0.42 | 0.987650 | 0.80 |
| 2 | 9 | 637384 | 0.29 | 5.13 | 0 | 2165120 | 1.13 | 0.68 | 1 | 0 |
| 3 | 6 | 29301 | 0.18 | 4.99 | 48 | 601464 | -0.85 | -0.72 | 1 | 0 |
| 4 | 11 | 534596 | 0.56 | 4.83 | 0 | 3370344 | -0.29 | NA | 0.999999 | 0.27 |
| 5 | 15 | 635439 | 0.60 | 5.13 | 322 | 4908800 | 0.95 | 0.68 | 0.992655 | 0.40 |
| 6 | 11 | 285059 | 0.58 | 5.03 | 0 | 1929850 | 0.55 | -0.17 | 0.999984 | 0.27 |
| 7 | 5 | 9126 | 0.14 | 4.64 | 0 | 206394 | -0.91 | -1.18 | 1 | 0 |
| 8 | 10 | 857381 | 0.39 | 5.13 | 48 | 4274582 | 1.76 | 0.22 | 0.999112 | 0.20 |
| 9 | 9 | 685234 | 0.34 | 5.12 | 0 | 2461968 | 1.26 | 0.22 | 0.999823 | 0.11 |
| 10 | 14 | 562613 | 0.75 | 4.83 | 0 | 3925386 | -0.09 | NA | 0.999998 | 0.43 |
| 11 | 11 | 285978 | 0.60 | 5.03 | 2 | 1997630 | 0.57 | -1.10 | 0.9999995 | 0.36 |
| 12 | 11 | 285974 | 0.57 | 5.05 | 139 | 3712604 | 0.57 | 1.69 | 1 | 0.09 |
| 13 | 14 | 98536 | 0.46 | 5.07 | 140 | 2410196 | -0.62 | 1.61 | 0.9999993 | 0.21 |
| 14 | 12 | 75957 | 0.59 | 4.93 | 0 | 1068178 | -1.73 | -0.17 | 0.999987 | 0.33 |
| 15 | 2 | 21801 | 0.01 | 4.76 | 0 | 27440 | -0.73 | -1.25 | 1 | 0 |
| 16 | 3 | 8933 | 0.03 | 4.56 | 0 | 362288 | -0.79 | -0.42 | 1 | 0 |
| 17 | 7 | 187826 | 0.09 | 1.03 | 185 | 629572 | 0.74 | NA | 0.999996 | 0.43 |
| 18 | 5 | 157431 | 0.02 | 1.02 | 0 | 380462 | -0.02 | NA | 1 | 0.20 |
| 19 | 14 | 523412 | 0.72 | 4.82 | 0 | 3363138 | -0.37 | NA | 0.999998 | 0.43 |
| 20 | 4 | 1947 | 0.25 | 3.84 | 0 | 39058 | -0.82 | -1.25 | 0.998094 | 0.50 |
| 21 | 14 | 259328 | 0.75 | 5.09 | 0 | 5538250 | 0.10 | 0.76 | 0.996071 | 0.36 |
| 22 | 11 | 718623 | 0.65 | 4.84 | 576 | 5097388 | 1.02 | NA | 0.999991 | 0.27 |
| 23 | 6 | 13587 | 0.17 | 4.71 | 0 | 582338 | -0.90 | -0.72 | 1 | 0 |
| 24 | 11 | 545171 | 0.43 | 5.14 | 456 | 4311012 | 0.57 | 0.22 | 0.979013 | 0.27 |
| 25 | 12 | 866186 | 0.61 | 4.84 | 406 | 5256294 | 2.10 | NA | 0.9999998 | 0.33 |
| 26 | 11 | 308742 | 0.41 | 1.72 | 0 | 967204 | -0.47 | NA | 0.999999 | 0.64 |
| 27 | 12 | 485849 | 0.64 | 4.82 | 0 | 2870498 | -0.64 | NA | 0.9999999 | 0.33 |
| 28 | 9 | 49434 | 0.26 | 5.00 | 96 | 966150 | -0.78 | 0.68 | 1 | 0 |
| 29 | 18 | 672839 | 0.83 | 5.16 | 481 | 21789038 | 1.32 | -0.42 | 0.927977 | 0.83 |
| 30 | 16 | 464640 | 0.81 | 5.15 | 187 | 2555564 | 1.19 | 1.25 | 0.999986 | 0.69 |
| 31 | 11 | 203183 | 0.60 | 5.06 | 0 | 3869496 | -0.47 | -1.10 | 0.996246 | 0.36 |
| 32 | 8 | 250309 | 0.43 | 5.06 | 0 | 1349522 | 0.17 | -1.10 | 0.999989 | 0.13 |
| 33 | 6 | 51629 | 0.28 | 5.10 | 0 | 648176 | -0.60 | 1.25 | 1 | 0.17 |
| 34 | 5 | 207110 | 0.03 | 1.02 | 48 | 611428 | 1.24 | NA | 1 | 0.20 |
| 35 | 6 | 282195 | 0.20 | 1.72 | 0 | 842352 | -0.81 | NA | 0.9999998 | 0.33 |
| 36 | 5 | 117706 | 0.02 | 1.02 | 0 | 238466 | -1.03 | NA | 1 | 0.20 |
| 37 | 4 | 5016 | 0.09 | 4.37 | 0 | 318044 | -0.92 | -1.65 | 1 | 0 |
| 38 | 16 | 323363 | 0.63 | 5.11 | 271 | 6536906 | 0.09 | 1.15 | 0.999556 | 0.38 |
| 39 | 13 | 261543 | 0.63 | 5.10 | 407 | 6885894 | 0.06 | -0.17 | 0.992450 | 0.38 |
| 40 | 12 | 134351 | 0.60 | 5.00 | 49 | 2699628 | -1.09 | -0.17 | 0.999998 | 0.33 |
| 41 | 9 | 165932 | 0.45 | 5.03 | 0 | 1097332 | -0.74 | -0.17 | 1 | 0.11 |
| 42 | 14 | 602442 | 0.76 | 4.83 | 0 | 3860468 | 0.20 | NA | 0.9999995 | 0.43 |
| 43 | 12 | 424142 | 0.62 | 4.81 | 0 | 2831282 | -1.08 | NA | 0.999999 | 0.33 |
| 44 | 8 | 440948 | 0.21 | 1.73 | 390 | 1156036 | 1.21 | NA | 0.999999 | 0.50 |
| 45 | 9 | 419961 | 0.31 | 1.73 | 0 | 995586 | 0.95 | NA | 0.9999999 | 0.56 |
| 46 | 9 | 456951 | 0.52 | 4.82 | 0 | 2863814 | -0.84 | NA | 1 | 0.11 |
| 47 | 6 | 53891 | 0.24 | 5.02 | 0 | 347492 | -0.78 | -1.18 | 0.991413 | 0.17 |
| 48 | 5 | 121940 | 0.02 | 1.02 | 0 | 257780 | -0.92 | NA | 1 | 0.20 |
| 49 | 12 | 418455 | 0.60 | 5.08 | 346 | 5283030 | 2.02 | 1.69 | 0.999999 | 0.17 |
| 50 | 7 | 276191 | 0.10 | 1.72 | 0 | 793808 | -0.88 | NA | 1 | 0.43 |
| 51 | 4 | 34878 | 0.09 | 4.84 | 97 | 1225704 | -0.67 | 0.42 | 1 | 0 |

**R code for supplementary example**

```
#make sure to set working directory#

#load igraph and tnet and ensure that the sna package is not currently loaded (sna and igraph interfere with each other)
detach("package:sna", unload=TRUE)
require(igraph)
require(tnet)

#read in association matrix and adjust from csv version
mat<-read.csv("Associationmatrix.csv")
mat<-as.matrix(mat[,2:ncol(mat)])

#convert into igraph object
#g is the weighted network and g2 the unweighted (binary) network
g<-graph.adjacency(mat,mode="undirected",weighted=T)
g2<-graph.adjacency(sign(mat),mode="undirected")

#plot the unweighted network to check that it has loaded properly
plot(g2)
dev.new()
plot.igraph(g2,vertex.size=8,vertex.label=NA,vertex.color="dark blue",edge.color="grey")

#calculate strength from the association matrix (by summing colums)
strength<-colSums(mat)

#calculate degree from the unweighted igraph network
deg<-degree(g2)

#calculate eigenvector centrality from the weighted igraph network
eigenvector<-evcent(g)

#create network as a tnet object (edgelist)
g3<-as.tnet(mat)

#calculate betweenness and closeness from tnet object
betweenness<-betweenness_w(g3)
closeness<-closeness_w(g3)

#load sna R package to calculate flow betweenness
library(sna)
#calculate flow betweenness
flow.betweenness<-flowbet(mat)

#-------------------------------------------------------------
#Calculate population-level metrics

#density from association matrix
density<-sum(mat>0)/(dim(mat)[1]*dim(mat)[2]-dim(mat)[1])
#unweighted transitivity in igraph
tr<-transitivity(g2)
#weighted transitivity in tnet
tr2<-clustering_w(g3)
#shortest pathlength in igraph
mean_distance(g2)


#-------------------------------------------------------------
#Modularity analysis
#determine community structure using fast.greedy algorithm in igraph
communities<-fastgreedy.community(g)
#and use to create a vector of community memberships
community.memberships<-membership(communities)

#---------------------------------------------------------------

#create Figure A in Box 1 of paper
library("RColorBrewer")
display.brewer.all()
pal<-brewer.pal(6,"Set1")
colours<-rep(NA,51)
for(i in 1:51){
colours[i]<-pal[community.memberships[i]]
}

sizes<-log(flow.betweenness)-4
par(mar=c(0,0,0,0))
plot.igraph(g2,vertex.size=(sizes),vertex.label=seq(1:51),vertex.label.cex=0.7,vertex.label.color="black",vertex.color=colours,edge.color="grey",vertex.frame.color=NA)

#--------------------------------------------------------------
#Calculate modularity metrics Zi and Pi

#sets up necessary vectors for input and to store results
indivs<-colnames(mat)
socgroups<-community.memberships
IGS<-rep(NA,length(indivs))
OGS<-rep(NA,length(indivs))
IGD<-rep(NA,length(indivs))
OGD<-rep(NA,length(indivs))
Zi<-rep(NA,length(indivs))
Zi.d<-rep(NA,length(indivs))

#calculate in and out of community strength for all individuals
for(i in 1:length(indivs)){
IGS[i]<-sum(mat[which(community.memberships%in%community.memberships[i]==TRUE),i])
OGS[i]<-sum(mat[which(community.memberships%in%community.memberships[i]==FALSE),i])
}

#calculate in and out of community degree for all individuals
for(i in 1:length(indivs)){
IGD[i]<-sum(mat[which(community.memberships%in%community.memberships[i]==TRUE),i]>0)
OGD[i]<-sum(mat[which(community.memberships%in%community.memberships[i]==FALSE),i]>0)
}

#calculate Pi from formula 2 in the main text
Pi<-1-(OGS/(IGS+OGS))^2
Pi.d<-1-(OGD/(IGD+OGD))^2

#calculate Zi using formula 1 from the main text
for(i in 1:length(indivs)){
Zi[i]<-(IGS[i]-mean(IGS[which(community.memberships%in%community.memberships[i]==TRUE)]))/sd(IGS[which(community.memberships%in%community.memberships[i]==TRUE)])
Zi.d[i]<-(IGD[i]-mean(IGD[which(community.memberships%in%community.memberships[i]==TRUE)]))/sd(IGD[which(community.memberships%in%community.memberships[i]==TRUE)])
}

#create a dataframe of all individual-level metrics
output<-data.frame(deg,strength,unlist(eigenvector$vector),closeness[,3]*10^6,betweenness[,2],flow.betweenness,Zi,Zi.d,Pi,Pi.d)

#read in TB data
TB.end<-read.csv("TBstats.csv")
TB.end[,2]<-factor(TB.end[,2],levels=c("N","P"))

#Create Figure B in Box 1
dev.new()
par(mar=c(5, 4, 4, 2),mfrow=c(1,2))
boxplot(deg~TB.end[,2],col="light blue",border="dark blue",las=1,ylab="Degree",xlab="Infection status",names=c("Uninfected","Infected"))
boxplot(Pi.d~TB.end[,2],col="light blue",border="dark blue",las=1,ylab=expression('P'[i]),xlab="Infection status",names=c("Uninfected","Infected")
```
