# Supplementary material for: Using Social Network Measures in Wildlife Disease Ecology, Epidemiology, and Management
Source: Bioscience. 2017 Feb 1;67(3):245–57. doi: 10.1093/biosci/biw175 (PMC5384163; doi:10.1093/biosci/biw175)
Supplement: Supplementary Data [file biw175_Supplementary_Data.zip › biw175_Suppp.zip!/Table S1.docx]

| **A** | | | | **B** | | | | **C** | | | | **D** | | | | **E** | | | | **F** | | | | **G** | | | |
| --- | --- | --- | --- | --- | --- | --- | --- | --- | --- | --- | --- | --- | --- | --- | --- | --- | --- | --- | --- | --- | --- | --- | --- | --- | --- | --- | --- |
|  | | | |  | | | |  | | | |  | | | |  | | | |  | | | |  | | | |
| ID | D | BC | FB | ID | D | BC | FB | ID | D | BC | FB | ID | D | BC | FB | ID | D | BC | FB | ID | D | BC | FB | ID | D | BC | FB |
| A | 5 | 0 | 4 | A | 4 | 0 | 2.4 | A | 3 | 0.25 | 3.5 | A | 2 | 0 | 1 | A | 2 | 6 | 12 | A | 8 | 0 | 7 | A | 15 | 0 | 14 |
| B | 5 | 0 | 4 | B | 5 | 0.25 | 4.7 | B | 4 | 0.667 | 5.167 | B | 2 | 0 | 1 | B | 1 | 0 | 0 | B | 8 | 0 | 7 | B | 15 | 0 | 14 |
| C | 5 | 0 | 4 | C | 5 | 0.25 | 4.7 | C | 5 | 0.917 | 5.667 | C | 3 | 6 | 13 | C | 1 | 0 | 0 | C | 8 | 0 | 7 | C | 15 | 0 | 14 |
| D | 5 | 0 | 4 | D | 5 | 0.25 | 4.7 | D | 4 | 0.667 | 5.167 | D | 3 | 6 | 13 | D | 2 | 4 | 8 | D | 8 | 0 | 7 | D | 15 | 0 | 14 |
| E | 5 | 0 | 4 | E | 5 | 0.25 | 4.7 | E | 4 | 0.25 | 3 | E | 2 | 0 | 1 | E | 2 | 6 | 2 | E | 8 | 0 | 7 | E | 15 | 0 | 14 |
| F | 5 | 0 | 4 | F | 4 | 0 | 2.4 | F | 4 | 0.25 | 3 | F | 2 | 0 | 1 | F | 2 | 4 | 8 | F | 8 | 0 | 7 | F | 15 | 0 | 14 |
|  |  |  |  |  |  |  |  |  |  |  |  |  |  |  |  |  |  |  |  | G | 8 | 0 | 7 | G | 15 | 0 | 14 |
|  |  |  |  |  |  |  |  |  |  |  |  |  |  |  |  |  |  |  |  | H | 8 | 0 | 7 | H | 15 | 0 | 14 |
|  |  |  |  |  |  |  |  |  |  |  |  |  |  |  |  |  |  |  |  | I | 8 | 0 | 7 | I | 15 | 0 | 14 |
|  |  |  |  |  |  |  |  |  |  |  |  |  |  |  |  |  |  |  |  |  |  |  |  | J | 15 | 0 | 14 |
|  |  |  |  |  |  |  |  |  |  |  |  |  |  |  |  |  |  |  |  |  |  |  |  | K | 15 | 0 | 14 |
|  |  |  |  |  |  |  |  |  |  |  |  |  |  |  |  |  |  |  |  |  |  |  |  | L | 15 | 0 | 14 |
|  |  |  |  |  |  |  |  |  |  |  |  |  |  |  |  |  |  |  |  |  |  |  |  | M | 15 | 0 | 14 |
|  |  |  |  |  |  |  |  |  |  |  |  |  |  |  |  |  |  |  |  |  |  |  |  | N | 15 | 0 | 14 |
|  |  |  |  |  |  |  |  |  |  |  |  |  |  |  |  |  |  |  |  |  |  |  |  | O | 15 | 0 | 14 |
|  |  |  |  |  |  |  |  |  |  |  |  |  |  |  |  |  |  |  |  |  |  |  |  | P | 15 | 0 | 14 |

**Table S1. The relationship between degree, flow betweenness and betweenness in simple toy networks.** Key to table: ID = individual in toy network (the ID individuals is alphabetical as you go round the circle, starting at the top), D = binary degree, BC = Freeman betweenness centrality, FB = flow betweenness
